# Sirtuin 4 Inhibits Prostate Cancer Progression and Metastasis by Modulating p21 Nuclear Translocation and Glutamate Dehydrogenase 1 ADP-Ribosylation

**DOI:** 10.1155/2022/5498743

**Published:** 2022-07-07

**Authors:** Liang Mao, Xi Hong, Luwei Xu, Xinning Wang, Jingyu Liu, Hao Wang, Yiguan Qian, Jun Zhao, Ruipeng Jia

**Affiliations:** ^1^Department of Urology, Nanjing First Hospital, Nanjing Medical University, Nanjing, Jiangsu, China; ^2^Department of Urology, The Affiliated Hospital of Qingdao University, Qingdao, Shandong, China

## Abstract

Protein posttranslational modification regulates several biological mechanisms, including tumor progression. In this study, we show that the mitochondrial Sirtuin 4 (SIRT4), which has ADP-ribosylation activity, plays a role in prostate cancer (PCa) progression. Firstly, SIRT4 expression was verified in PCa tissues and cell lines by quantitative real-time PCR (qRT-PCR) and western blotting. Subsequently, we established stable PC-3 and 22rv1 cells that overexpressed SIRT4 and knocked down SIRT4, respectively. The functions of SIRT4 in PCa were explored through various phenotype experiments. The mechanism underlying the functions of SIRT4 was investigated through western blotting, immunoprecipitation, immunofluorescence, and nuclear and cytoplasmic extraction assays. We revealed that SIRT4 inhibited cell progression both in vivo and in vitro. Mechanistically, on the one hand, SIRT4 promoted the ADP-ribosylation of glutamate dehydrogenase 1 to inhibit the glutamine metabolism pathways. On the other hand, SIRT4 inhibited the phosphorylation of AKT, thereby affecting p21 phosphorylation and its cellular localization for cell cycle arrest. In conclusion, our study indicates that SIRT4 is directly associated with PCa progression and could be a novel target for PCa therapy.

## 1. Introduction

The incidence of prostate cancer (PCa) has surpassed that of lung and bronchus cancer to become the most common cancer that affects elderly men worldwide [1]. Likewise, in China, the incidence of PCa has increased over the years. Several PCa patients progress to castration-resistant prostate cancer (CRPC) at an advanced stage; thus, early diagnosis and treatment for PCa patients are needed [2]. Accordingly, an investigation into the molecular mechanisms underlying PCa progression is urgently required.

The abnormal metabolic characteristics of tumors are considered a hallmark of cancer. Recently, more studies have revealed that the enzymes that play a key role in tumor metabolism could be modulated by numerous posttranslational modifications (PTMs), including acetylation, methylation, ubiquitination, succinylation, and crotonylation [3, 4]. As a member of the sirtuin family, sirtuin 4 (SIRT4) exhibits deacetylation and ADP-ribosylation activities [5, 6]. Initially, research on SIRT4 was mainly focused on cellular metabolisms, such as insulin secretion and fatty acid oxidation; however, currently, there is an increased focus on its role in cancer. Accumulating evidence has demonstrated that SIRT4 suppresses tumor progression by inhibiting glutamine metabolism. Glutamine plays a crucial role in tumor metabolism, especially in PCa, because the growth of this cancer is glutamine-dependent [7]. Therefore, we speculated that SIRT4 plays a biological role in PCa development.

Glutamate dehydrogenase (GDH), a key enzyme, contributes to glutamine metabolism. GDH has two isoforms in humans, glutamate dehydrogenase 1 (GLUD1) and glutamate dehydrogenase 2 (GLUD2), with the former expressed universally and the latter found substantially in the brain, testis, and kidney [8–12]. GLUD1 has been shown to localize to the mitochondria at the cellular level, and its primary function is to convert glutamic acid to *α*-ketoglutarate (*α*-KG) [13]. In human cancer cells, GLUD1 expression is generally elevated to ensure that the cells produce energy for rapid growth.

P21 is a well-known protein that could regulate the cell cycle [14, 15]. It also plays an essential role in several cellular activities, such as cell migration and invasion, DNA damage repair, and apoptosis. Since changes in p21 expression have been reported in various cancers, it is deemed as an indicator of cancer [16]. However, recent studies have shown that p21 exerts tumor suppressor or tumor-promoting effects due to its different cellular localization [17, 18]. The accumulation of p21 in the nucleus can block the cell cycle by inhibiting CDK4, 6-cyclin D1 complex and CDK2-cyclin E complex formation, respectively [19].

In the present study, we aimed to reveal the biological functions of SIRT4 and its role in the development of PCa. The upregulation of SIRT4 inhibited proliferation, migration, and invasion ability of PCa cells, while SIRT4 downregulation facilitated these abilities. In addition, we found that SIRT4 regulated GLUD1 ADP-ribosylation and inhibited AKT phosphorylation. Our findings suggest that SIRT4 could be a promising therapeutic target for PCa.

## 2. Methods

### 2.1. Patients Information and Clinical Tissue Samples

The PCa tissues and the normal prostate tissues enrolled in this study were acquired from the Nanjing First Hospital affiliated with Nanjing Medical University and rigorously scored by the Department of Pathology from 2018 to 2020. All tissues were cryopreserved in liquid nitrogen. This study was approved by the Ethics Committee of Nanjing First Hospital affiliated with Nanjing Medical University. The basic clinical information of PCa patients is listed in Additional file 1: Table S1.

### 2.2. Cell Lines

Human normal prostate epithelial cell line (RWPE-1) and the human PCa cell lines (DU145, PC-3, PC-3M, 22rv1, and LNCaP) were purchased from the Cell Bank of Shanghai Chinese Academy of Science (Shanghai, China). 22rv1 cells were cultured in RPMI 1640 medium (Gibco, USA) with 10% fetal bovine serum (FBS) (Gibco, USA), 1% penicillin, and streptomycin (Gibco, USA). PC-3 cells were cultured in F-12K (Gibco, USA) with 1% penicillin, 1% streptomycin, and 10% FBS. All cells were cultured in an incubator with 5% CO_2_ and 95% humidity at 37°C.

### 2.3. RNA Extraction and qRT-PCR

According to the manufacturer's protocol, an RNA-easy isolation reagent (Vazyme, Suzhou, China) was used to extract total RNA from PCa cells. The relative mRNA expression was confirmed by using a SYBR Green PCR Kit (Takara, Japan) on an ABI 7500 real-time PCR system (Applied Biosystems, CA, USA). The gene expression level was calculated by using the 2^−ΔΔCT^ method. GAPDH expression was used as a control to standardize target gene expression in different RNA samples. The primer sequences are listed in Table S2.

### 2.4. Lentivirus Infection

The lentiviral short hairpin RNA (shRNA) constructs targeting SIRT4 was designed by Shanghai Genechem (Shanghai, China). The short hairpin RNA of SIRT4 was packaged into GV493 lentiviral vectors. In accordance with the manufacturer′s instructions, cells cultured in a complete medium were transduced with lentivirus at an appropriate multiplicity of infection and 30%–50% confluence (PC-3 cells, MOI = 50; 22rv1 cells, MOI = 5). After 2 days of transduction, cells were selected by using a complete medium that contains 2 *μ*g/ml puromycin for 14 consecutive days. All shRNA sequences are listed in Table S3.

### 2.5. Western Blotting

The proteins from PC-3 and 22rv1 cells were isolated by using a lysis buffer (KeyGEN, China). The protein samples were measured by bicinchoninic acid assay (KeyGEN, China). The samples were separated by SDS-PAGE and transferred to PVDF membranes (Millipore, USA). The membranes were blocked with 5% skim milk (BioFrox, Guangzhou) and then incubated with appropriate primary antibodies diluted in 3% BSA at 4°C overnight. Then, the membranes were incubated with peroxidase-conjugated secondary antibodies for 1 h at room temperature. Proteins were visualized using the ECL Plus bioimaging system (Millipore, Billerica, MA, USA). Actin was used as the internal reference. The information and dilution ratios of antibodies are listed in Table S4.

### 2.6. Immunofluorescence

A suitable number of SIRT4 overexpression cells was added to 24-well plates, cultured in a complete medium, fixed in 4% methanol, and treated with 1% Triton-100 on ice. Next, each well was blocked with 5% BSA containing 0.2% Triton-100. After washing with PBST three times, the wells were cultured with the primary antibody at 4°C overnight. Next, the plate was incubated with the fluorescent secondary antibody at 37°C for 1 h in the dark. The images of the cells were acquired using a fluorescence microscope (×200) (Olympus, Japan).

### 2.7. CCK-8 and Colony Formation Assay

Five thousand lentivirus-infected cells were seeded into 96-well plates, and three parallel wells were set up for each group. CCK8 reagent (Beyotime, China), at a volume of 10 *μ*l, was added into a 90 *μ*l complete medium in each well at the following time points: 0, 1, 2, 3, and 4 days. The cell multiplication capacity was determined according to the OD value at 450 nm using a microplate reader (TWIN200PRO, TECAN, Swiss). Approximately 500 lentivirus-infected cells were plated into each well of 6-well plates and cultured in a complete medium for 2 weeks continuously. Then, the cells were fixed in 4% paraformaldehyde and stained with 5% crystal violet (Beyotime, China). The number of colonies was counted under a microscope (NIKON TI, Tokyo, Japan).

### 2.8. Transwell Assay

After overexpression or knockdown of SIRT4, the cells were added into the upper chambers of transwell plates (Corning, USA) containing 200 *μ*l of serum-free medium. For the invasion experiment, the upper chambers were covered with Matrigel (BD Biosciences, USA). The lower chambers contained a 600 *μ*l culture medium with 10% FBS. After 72 h of cultivation in an incubator, cells from the upper chamber were removed by using a cotton swab. Those cells that passed through the pores to the lower chambers were fixed in 4% paraformaldehyde and stained with 5% crystal violet. Next, cells in three fields of view were counted and imaged using an FSX100 microscope (Olympus, Japan).

### 2.9. Wound-Healing Assay

The lentiviral-infected cells were seeded into 6-well plates and incubated in a humidified 5% CO_2_ incubator when their confluence was approximately 90%. Wounds were created using a 10*μ* plastic pipette tip. The images were taken at 0 h and 24 h after wounding using an FSX100 microscope (Olympus, Japan). ImageJ software was used to measure the scratched areas.

### 2.10. Nuclear and Cytoplasm Extraction

The cytoplasmic and nuclear proteins were separated using a NE-PER Nuclear and Cytoplasmic Extraction Kit (Thermo Fisher Scientific, USA) following protocol. Cells were harvested and the supernatant was discarded cautiously to make the microcentrifuge tube as dry as possible. Next, some specific reagents provided in the kit were added into the tubes to gain nuclei and cytoplasm protein. The proteins extracted from these components were analyzed by western blotting. The laminB1 and GAPDH antibody were used as nuclear protein internal reference and cytoplasmic protein marker, respectively.

### 2.11. Immunoprecipitation

PC-3 and 22rv1-SIRT4 overexpression cells were cultured in 75 cm^2^ culture bottles and lysed in 1 ml IP lysis buffer with PMSF, protease inhibitors, and phosphatase inhibitors on ice for 15 min. The supernatant was collected and divided into three groups: input, IgG, and IP. The IP group was treated with the target antibody (4–8 *μ*g), and the IgG group was treated with the anti-mouse/rabbit IgG antibody (4–8 *μ*g); both groups were incubated at 4°C. The following day, 40 *μ*l of protein A/G magnetic beads washed three times by PBST were added to the antigen-antibody complex-containing solution and placed on a shaker at 4°C. Then, the beads were collected with a magnetic rack, and a 40 *μ*l elution buffer was added. The mixture was incubated for 30 min at room temperature. The supernatant was collected, and 8 *μ*l of a neutralization buffer was added immediately. IP proteins were analyzed by western blotting after adding 5× SDS loading buffer.

### 2.12. Tumor Xenograft Formation Experiment

Ten male BALB/c nude mice aged 5–6 weeks were maintained under specific pathogen-free conditions for this experiment. The infected PC-3 cells were suspended in PBS and injected into the left and right flanks of the mice to induce tumor formation. The volume of the tumor was calculated by the following formula: (*L* × *W*^2^)/2, where *L* = length and *W* = width, respectively. The length and width of the tumor were measured every 6 days for 1 month. Finally, the nude mice were sacrificed, and the weights and volumes of the tumors were determined. Tumor tissues were fixed in paraformaldehyde (Beyotime, China) for hematoxylin and eosin (HE) staining and immunohistochemistry.

### 2.13. Statistical Analysis

The statistics were analyzed using SPSS 19.0 (Statistical Product and Service Solutions). The differences between the two groups were conducted by Student's *t*-test, while comparisons among more than two groups were performed using one-way ANOVA. *P* < 0.05 was deemed as statistically significant. All data are presented as the mean ± standard deviation (SD) from at least three independent replicates.

A complete description of the methods, including 5-ethynyl-2′-deoxyuridine (EdU) assay, immunohistochemistry (IHC), and flow cytometry assay is available in Additional file 2: Supplemental materials and methods (available here).

## 3. Results

### 3.1. SIRT4 Is Downregulated in PCa

**Figure 1:**
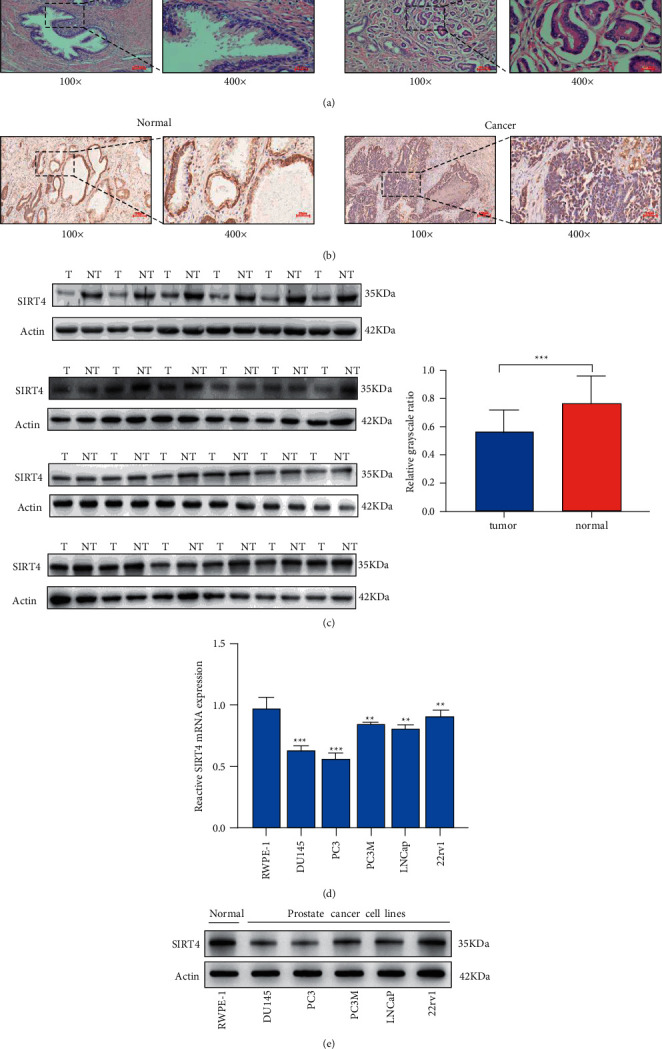
SIRT4 expression is downregulated in PCa. (a) The results of HE staining. (b) SIRT4 protein expression was detected by immunohistochemistry in paired of PCa tissue and adjacent tissue. (c) SIRT4 protein expression was analyzed by western blotting in 24 pairs of PCa tissues and prostate normal tissues. The results were measured by grayscale analysis. (d, e) The qRT-PCR and western blotting were used to detect the SIRT4 expression in n a normal prostate epithelial cell line (RWPE-1), and prostate cancer cell lines (DU145, PC-3, PC-3M, 22rv1, and LNCap), respectively, magnification ×100, scale bar = 100 *μ*m, magnification ×400, scale bar = 25 *μ*m ^*∗*^*P* < 0.05^*∗∗*^*P* < 0.01, ^*∗∗∗*^*P* < 0.001.

Several studies have reported that SIRT4 has a low expression in tumors, such as colorectal cancer, and clear cell renal cell carcinoma [20, 21]. To evaluate SIRT4 expression in PCa, several data analyses and experiments were performed. First, according to the previous study [22], SIRT4 has a lowly expressed in PCa tissue samples. As shown in Figure 1(a), PCa was observed by H&*E* staining. Based on immunohistochemistry results of PCa and normal prostate tissues, we found that the SIRT4 had a low expression in tumors (Figure 1(b)). Subsequently, we analyzed SIRT4 protein expression in 24 pairs of PCa tissues by western blotting. The results proved that SIRT4 was expressed at low levels in clinical PCa tissues compared to normal prostate tissues (Figure 1(c)). To better understand SIRT4 protein expression in PCa cell lines, we performed qRT-PCR and western blotting experiments. To this end, we selected the normal epithelial prostate cell line RWPE-1 and five PCa cell lines DU145, PC-3, PC-3M, 22rv1, and LNcaP. All five PCa cell lines indicated lower SIRT4 expression than normal epithelial prostate cell lines. Furthermore, qRT-PCR results demonstrated that SIRT4 mRNA was downregulated in PCa cells. Thus, we conclude that SIRT4 expression is downregulated in PCa. The clinical and pathological features of PCa patients are listed in Table 1.

### 3.2. SIRT4 Inhibits the Proliferation, Migration, and Invasion Ability of PCa Cells

**Figure 2:**
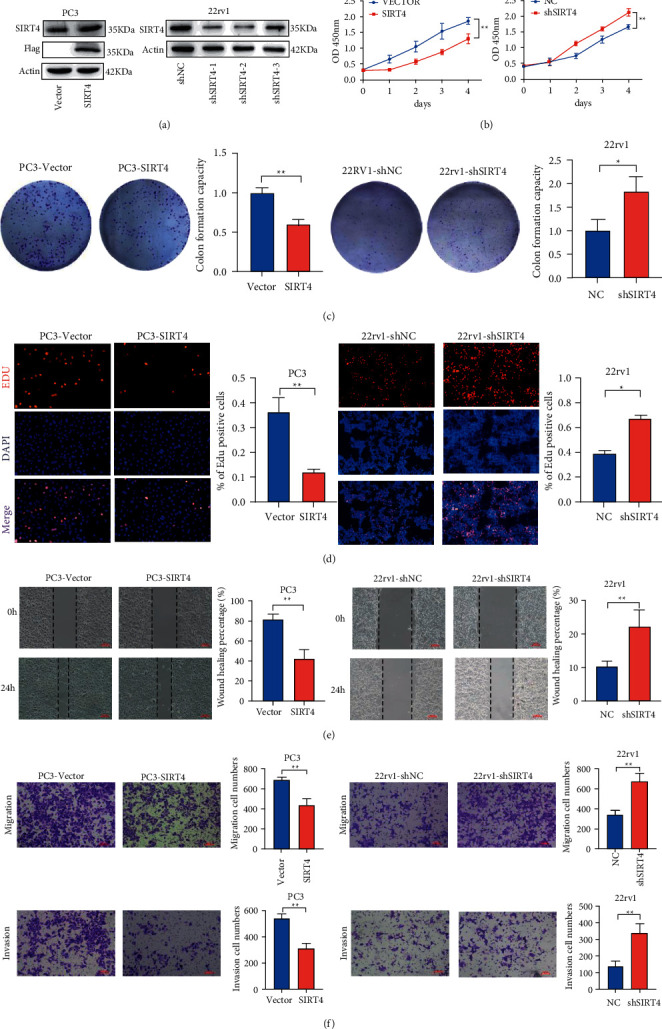
SIRT4 inhibits proliferation and cell cycle in PCa cells. (a) The SIRT4 protein expression levels in SIRT4-overexpressing PC-3 (left) and SIRT4-knockdown 22rv1 (right) cells were detected by western blotting. (b) CCK8 assay. The effect of SIRT4 overexpression or silencing on the proliferation of PC-3 and 22rv1. (c) Colony formation assay SIRT4-overexpressing cells and SIRT4-silenced cells were seeded onto 6-plates, and the number of colonies was counted on day 14. (d) EdU assay was used to detect proliferation ability in PC-3 and 22RV1 cells. (e) Wound-heal assay was detected at 0 h and 24 h to determine cell migration ability in SIRT4-overexpressing and SIRT4-knockdown PCa cells. (f) Transwell assays were used to determine the invasion and migration abilities of SIRT4-overexpressing and SIRT4-knockdown PCa cells. Scale bar = 100 *μ*m ^*∗*^*P* < 0.05^*∗∗*^*P* < 0.01, ^*∗∗∗*^*P* < 0.001.

The specific function of SIRT4 in PCa was rarely reported in previous studies. We overexpressed SIRT4 in PC-3 cells and knocked it down in 22rv1 cells, and its infection efficiency was determined by western blotting (Figure 2(a)). Simultaneously, we conducted several phenotypic experiments related to tumors to verify whether SIRT4 affects tumor progression.

CCK-8 assay results demonstrated that SIRT4 overexpression suppressed PC-3 cell proliferation, and SIRT4 knockdown promoted 22rv1 cell growth (Figure 2(b)). Similarly, colony formation and EdU assays also showed that SIRT4 overexpression inhibited cell proliferation, while SIRT4 knockdown promoted cell proliferation (Figures 2(c) and 2(d)). Then, we detected the effect of SIRT4 on the migration and invasion ability in PC-3 and 22rv1 cells. As shown in Figure 2(e), the wound-healing assay showed that SIRT4 silencing promoted the healing of scratch wounds, while SIRT4 overexpression prolonged wound closure. Transwell assay results confirmed that SIRT4 knockdown accelerated the migration and invasion ability of 22rv1 cells. In contrast, reduced abilities of migration and invasion were observed in SIRT4-overexpressing PC-3 cells (Figure 2(f)). Altogether, these findings suggest that SIRT4 affects PCa cell proliferation, migration, and invasion. Thus, based on these results, SIRT4 plays an antitumor role in PCa cells.

### 3.3. SIRT4 Overexpression Impairs Tumor Growth Ability *In Vivo*

**Figure 3:**
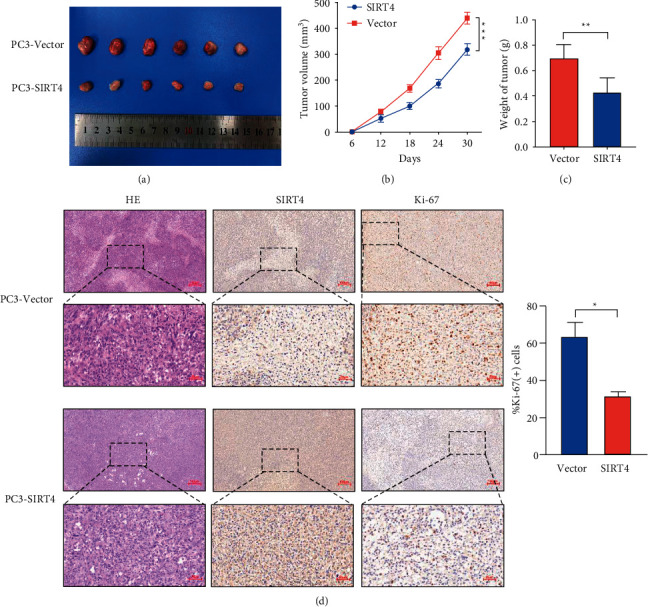
SIRT4 overexpression suppresses xenograft tumor growth in vivo. (a) Overexpression of SIRT4 dramatically inhibits subcutaneous tumor growth in nude mice. (b) The volumes of xenograft tumors were measured by the vernier caliper every 6 days. (c) The weight of tumors in the PC-3-Vector and PC-3-SIRT4 groups. (d) The results of HE staining and IHC indicate that overexpression of SIRT4 reduced the expression of Ki-67. Magnification ×100, scale bar = 100 *μ*m, magnification ×400, scale bar = 25 *μ*m, ^*∗*^*P* < 0.05^*∗∗*^*P* < 0.01, ^*∗∗∗*^*P* < 0.001.

The purpose of constructing a xenograft tumor mouse model was to understand the function of SIRT4 in vivo. PC-3 cells infected with lentivirus-SIRT4, and lentivirus control were suspended with PBS and injected into nude mice subcutaneously. Subsequently, the volume of tumors was measured with calipers every 6 days. A month later, the tumors were collected, imaged, and weighed. We found that the tumor lumps in the SIRT4 overexpression group were markedly lower than that in the control group (Figures 3(a) and 3(b)). A line chart was drawn based on the volume of the tumor and the time of measurement. As shown in Figure 3(c), the rate of growth of the tumor acquired from the SIRT4 overexpression group was obviously slower than that obtained from the control group. In addition, in the SIRT4-overexpressing group, the average tumor weight was also lower than that in the control group. Morphological variation of the tumor was observed by HE staining and immunohistochemistry. The SIRT4 overexpression group had a lower Ki-67 positive rate (Figure 3(d)). These results demonstrate that SIRT4 inhibits cell growth in vivo, which is also consistent with its biological function in vitro.

### 3.4. SIRT4 Decreases the Phosphorylation of p21 through AKT Signaling to Induce Cell Cycle Arrest

**Figure 4:**
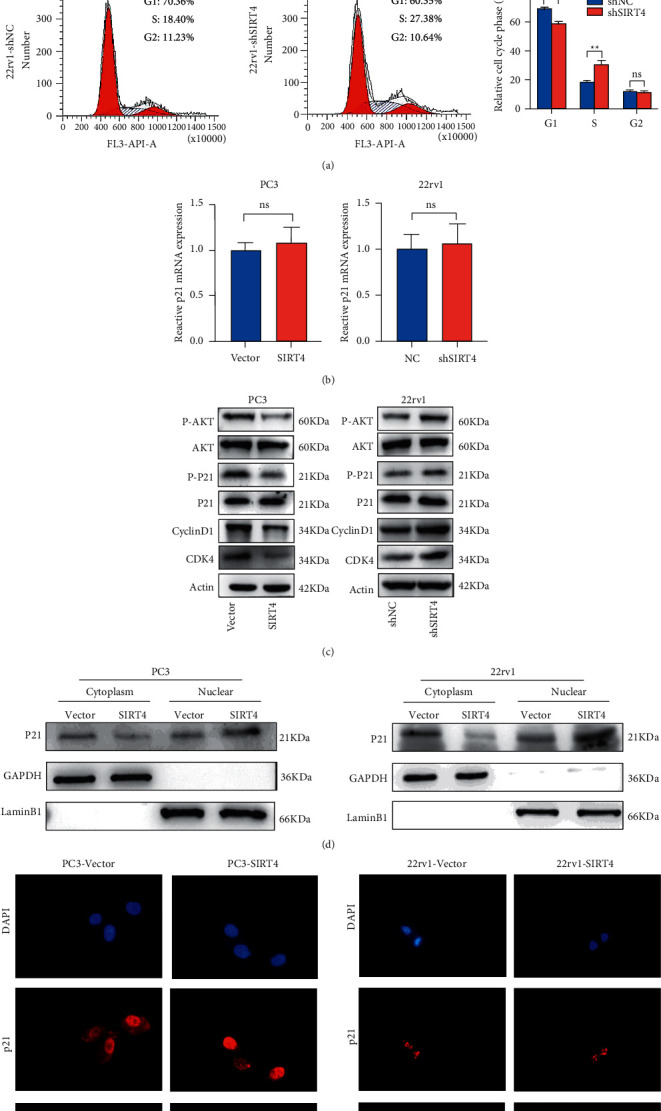
SIRT4 restrains AKT phosphorylation and promotes p21 nuclear localization. (a) The cell cycle of SIRT4-overexpressing and SIRT4-knockdown cells were detected by flow cytometry. (b) The mRNA expression of p21 in SIRT4-overexpressing and SIRT4-knockdown cells were contrasted by qRT-PCR. (c) The pivotal members (AKT, p-AKT, p21, p-P21, cyclin D1, and CDK4) protein levels were determined by western blotting in SIRT4-knockdown 22rv1 and SIRT4-overexpressing PC-3 cells. (d) Nuclear and cytoplasm extraction assay. The p21 protein expression of SIRT4-overexpressed PC-3 and 22rv1 cells in nuclear and cytoplasm was analyzed by western blotting, respectively. (e) Immunofluorescence was used to ensure the location of p21 more directly in SIRT4-overexpressing PC-3 and 22rv1 cells. Red fluorescence represents p21, and nuclear was stained by DAPI (blue).

It is widely recognized that p21 exerts a crucial role in many cellular processes of tumors, including cell cycle and proliferation ability [14, 23]. First, we applied flow cytometry cell cycle assays to detect cell cycle progression. These results proved that SIRT4 overexpression induced cell cycle arrest at the G1 phase (Figure 4(a)). Then, the qRT-PCR results demonstrated that SIRT4 overexpression did not affect the mRNA levels of p21 in PC-3 and 22rv1 cells (Figure 4(b)). Moreover, it is previously reported that p53 is not expressed in PC-3 cells [24]. Therefore, we speculated that SIRT4 might inhibit the cell cycle by affecting the phosphorylation of AKT. In our study, we used a western blotting experiment to detect the expression of proteins that were related to the AKT signaling pathway in PC-3 and 22rv1 cells. We discovered that the expression of p-AKT, p-P21, cyclin D1, and CDK4 decreased in SIRT4-overexpressing PC-3 cells and increased in SIRT4-silenced 22rv1 cells. However, it has no significant difference in total AKT and p21 protein levels in PC-3 and 22rv1 cells (Figure 4(c)). It has been reported that p21 inhibits the cell cycle because of its decreased phosphorylation and subcellular localization [25]. In the nucleus, p21 can block the cell cycle by inhibiting CDK4/cyclin D1 [19]. SIRT4 overexpression decreased the levels of p-p21 and promoted p21 nuclear translocation. To test this possibility, we overexpressed SIRT4 in both PC-3 and 22rv1 cells. Subsequently, we isolated nuclear and cytoplasmic proteins and found that p21 protein expression was increased in the nuclear extracts of SIRT4-overexpressing PC-3 cells compared with normal PC-3 cells (Figure 4(d)). Immunofluorescence experiments were also conducted to observe p21 nuclear accumulation in SIRT4 overexpression PC-3 cells (Figure 4(e)). Collectively, these results confirmed that SIRT4 could inhibit the phosphorylation of p21 and AKT and affect the cell cycle to suppress PCa cell proliferation.

### 3.5. SIRT4 Affects GLUD1 Expression through Its ADP-Ribosylation Activity, Inhibiting PCa Cell Proliferation, Migration, and Invasion

Among the differentially expressed proteins, GLUD1 is of attention for the following reasons. First, it is established that glutamine metabolism plays a crucial part in PCa growth [7]. Second, GLUD1 acts as a key metabolic factor. Glutamate is converted to *α*-KG by the action of GLUD1, and *α*-KG contributes to the tricarboxylic acid cycle and provides energy [13]. Moreover, SIRT4 could interact with GLUD1 to participate in regulating glutamine metabolism [26].

**Figure 5:**
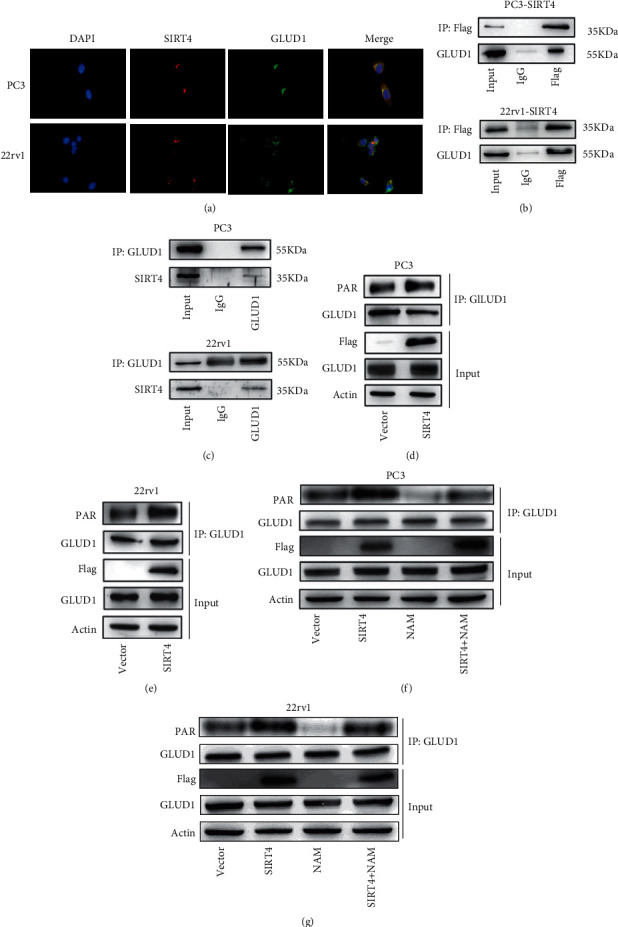
SIRT4 plays an inhibitory role in cancer by mediating the ADP-ribosylation of GLUD1. (a) Immunofluorescence experiments show that SIRT4 and GLUD1 proteins colocalize in PC-3 and 22rv1 cells. (b) The protein of SIRT4-overexpressing PC-3 and 22rv1 cells was immunoprecipitated with an anti-Flag antibody, and GLUD1 was analyzed by western blotting. GLUD1 was detected in the immunoprecipitated SIRT4 complex, not in the IgG sample. (c) The protein of PC-3 and 22rv1 cells was immunoprecipitated with GLUD1 antibody (rabbit), and SIRT4 was determined by western blotting. SIRT4 was detected in the immunoprecipitated GLUD1 complex, not in the IgG sample. (d, e) The GLUD1 protein was purified in PC-3-Vector, PC-3-SIRT4, 22rv1-vector, and 22rv1-SIRT4 cells, and the ADP-ribosylation of GLUD1 was analyzed by western blotting. (f, g) Endogenous GLUD1 in PC-3 and 22rv1 cells treated with nicotinamide (NAM, 5 mM, 6 h was immunoprecipitated with GLUD1 antibody. The ADP-ribosylation of GLUD1 was analyzed using an anti-PAR antibody via western blotting.

To expound on the molecular mechanism underlying the interaction between SIRT4 and GLUD1, we examined the colocalization of SIRT4 and GLUD1 using immunofluorescence. As shown in Figure 5(a), in which red fluorescence represents SIRT4 and green fluorescence GLUD1, SIRT4, and GLUD1 colocalized in the mitochondria. The direct interaction between SIRT4 and GLUD1 was further confirmed in both PC-3 and 22rv1 cells using a coimmunoprecipitation assay (Figures 5(b) and 5(c)). Western blotting results revealed that SIRT4 did not affect the expression of GLUD1. Considering that SIRT4 is mainly considered an ADP-ribosyltransferase, we further determined whether this interaction could affect the lysine ADP-ribosylation level of GLUD1 in PC-3 and 22rv1 cells. Interestingly, when SIRT4 was overexpressed, the APD-ribosylation level increased (Figures 5(d) and 5(e)). Finally, we demonstrated whether SIRT4 suppressed PCa cell progression and metastasis by regulating the modification of GLUD1. To further confirm the effect of SIRT4 inhibition, nicotinamide (NAM) was used to verify ADP-ribosylation levels. It binds to a conserved pocket adjacent to that of NAD^+^ to block the deacetylase and ADP-ribosyltransferase activities of SIRT4 [27]. As shown in Figures 5(f) and 5(g), nicotinamide decreased the level of ADP-ribosylation in PC-3 and 22rv1 cells. These data confirmed that SIRT4 exerted an inhibitory effect by combining with GLUD1 and affected the ADP-ribosylation of GLUD1.

### 3.6. Suppression of ADP-Ribosylation Reverses the Function of SIRT4 in PCa Cells

**Figure 6:**
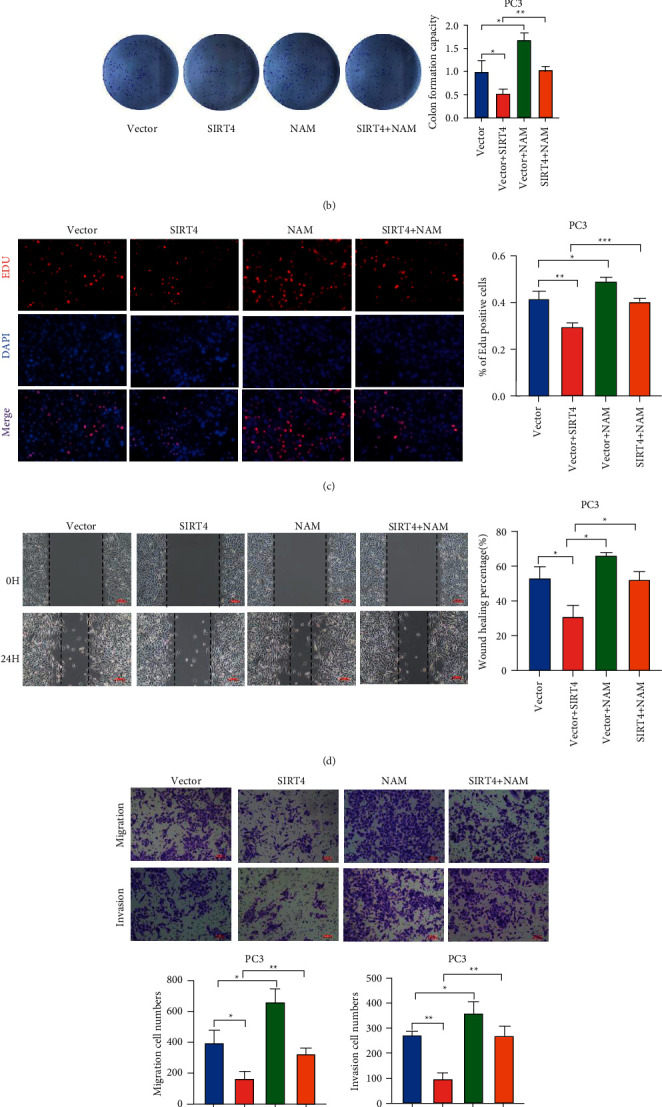
SIRT4 suppresses cell proliferation, migration, and invasion through the ADP-ribosylation of GLUD1. (a–c) Cell proliferation ability was analyzed by CCK8 assay, colony formation assay, EdU assay with PC-3, and SIRT4-overexpressing PC-3 cells with or without being treated with nicotinamide. (d) The cell migration of PC-3 and PC-3-SIRT4 cells was measured after dealing with or without nicotinamide by wound-healing assay. (e) Effects of SIRT4 on migration and invasion abilities were detected by Transwell assay. Scale bar = 100 *μ*m, ^*∗*^*P* < 0.05^*∗∗*^*P* < 0.01, ^*∗∗∗*^*P* < 0.001.

To detect whether SIRT4 plays a tumor-suppressive role through the modification of GLUD1 in PCa, we performed various rescue experiments. PC-3 cells were processed and divided into four groups: vector, SIRT4-overexpressing, NAM, and both. Similarly, we conducted a series of phenotype assays to examine cell proliferation ability. As shown in Figures 6(a) and 6(b), CCK-8 and colony formation assay results indicated that the ability of proliferation was weakened by overexpressing SIRT4 and could be reversed by adding NAM. EdU assay drew the same conclusion directly (Figure 6(c)). Wound healing and transwell assays showed a weakened migration and invasion ability due to SIRT4 overexpression, and this ability was reversed by the suppression of ADP-ribosylation (Figures 6(d) and 6(e)). In conclusion, our results provide evidence that SIRT4 inhibits PCa cell progression via the ADP-ribosylation of GLUD1.

### 3.7. AKT Inhibitor MK-2206 Suppresses the Functions of SIRT4 in PCa Progression

**Figure 7:**
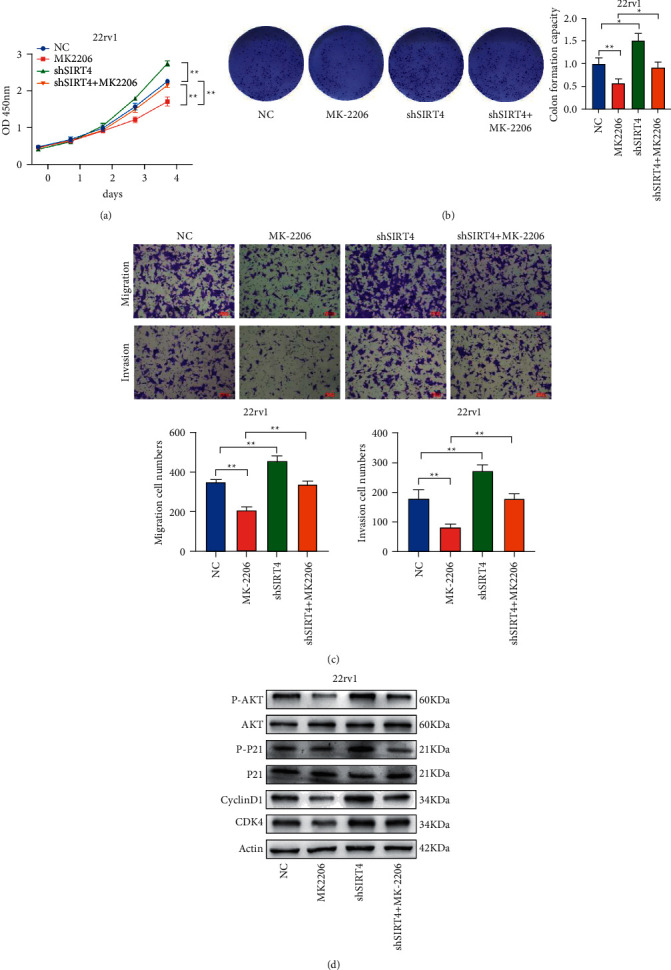
Rescue experiments proved that SIRT4 plays it inhibitory role by affecting the p21 phosphorylation. (a, b). CCK-8 and colony formation assays showed that the enhanced proliferation ability of the 22rv1-shSIRT4 cell was blocked after Akt inhibition. (c) The results of the transwell assay demonstrated that MK-2206 inhibited the migration and invasion ability of SIRT4 silencing 22rv1 cells. (d) Western blotting was used to indicate that MK-2206 inhibited the activation of p21 phosphorylation. Scale bar = 100 *μ*m, ^*∗*^*P* < 0.05^*∗∗*^*P* < 0.01, ^*∗∗∗*^*P* < 0.001.

**Figure 8:**
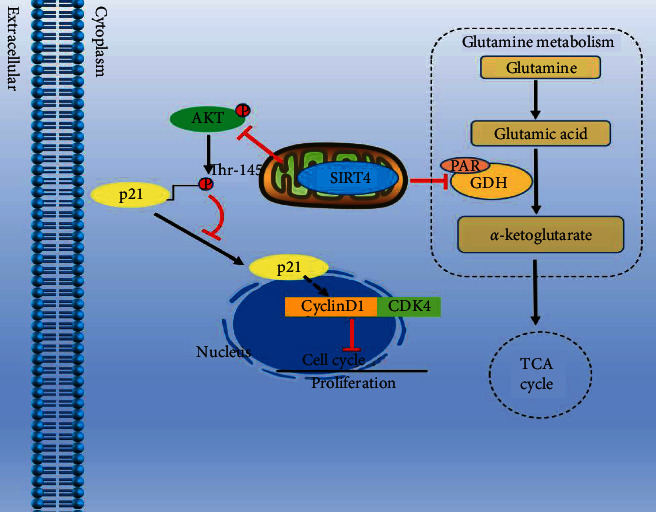
The schematic model shows that SIRT4 inhibits the proliferation, migration, and invasion of PCa cells in two ways, one is affecting p21 phosphorylation and its subcellular localization, and the other one is regulating glutamine metabolism by GLUD1 ADP-ribosylation.

To determine whether SIRT4 inhibits PCa cell proliferation, migration, and invasion by affecting p21 phosphorylation, we treated the control group and SIRT4 silencing group with AKT inhibitor MK-2206 and designed some rescue experiments. As shown in Figures 7(a) and 7(b), CCK-8 and colony formation experiments indicated that PCa cells proliferation and colony formation ability were restrained by AKT inhibitor MK-2206 and reversed by knocking-down of SIRT4. Transwell assays revealed that the attenuated migration and invasion abilities induced by MK-2206, were reversed by SIRT4 silencing in 22rv1 cells (Figure 7(c)). As indicated in Figure 7(d), SIRT4 silencing increased p-AKT, p-p21, cyclin D1, and CDK4 proteins expression without influencing the total protein levels of AKT and p21. MK-2206 reversed the activation of AKT signaling after SIRT4 knocking down in the 22rv1 cell. Taken together, the results clearly demonstrate that SIRT4 suppresses PCa progression via modulating the phosphorylation of p21. These findings suggested that SIRT4 regulated PCa progression in two ways, on the one hand, it could affect p21 localization to arrest the cell cycle, and on the other hand, it affects glutamine metabolism pathways (Figure 8).

## 4. Discussion

In the present study, we proved that SIRT4 plays a crucial role in PCa progression. We discovered that SIRT4 overexpression inhibited PCa cell progression and metastasis. Moreover, we observed that SIRT4 silencing promoted PCa cell proliferation by activating the cell cycle. In addition, we found that SIRT4 blocked the cell cycle by inhibiting the phosphorylation of AKT and p21. SIRT4 also interacts with GLUD1 and affects ADP-ribosylation modification in glutamine metabolism to suppress PCa cell proliferation, migration, and invasion.

Sirtuins are NAD^+^-dependent deacetylases that play significant roles in regulating cellular metabolism. SIRT4 is one of the three mitochondrial sirtuins and was first described as an ADP-ribosyltransferase [28]. It mediates the activity of normal and tumor cells by regulating different signaling pathways. It has been reported that SIRT4 is decreased in colorectal cancer (CRC) and exerts inhibitory effects by targeting GLS-mediated AKT/GS3*β*/cyclin D1 [20]. Further, SIRT4 can arrest the cell cycle and induce apoptosis in hepatitis B virus-related hepatocarcinoma [29]. Moreover, UHRF1 promotes cell proliferation through the suppression of SIRT4 in pancreatic cancer [30]. Csibi et al. also proved that SIRT4 inhibited the proliferation of human CRC DLD-1 cells and PCa DU145 cells. [31] However, its primary function and mechanism in PCa are unknown. In this study, we demonstrated that SIRT4 inhibits the malignant progression of PCa cells.

It is established that p21 can participate in the proliferation and cell cycle of several types of tumor cells [32]. Previous studies have reported that *δ*-catenin participates in EGF/AKT/p21^Waf^ signaling, inducing PCa cell proliferation and invasion [25]. In our study, we found that the cell cycle was blocked in the G1 phase, and p21 played a role in inhibiting the cell cycle through AKT phosphorylation. As a key factor involved in regulating the cell cycle, p21 has dual functions because of its subcellular localization. When p21 enters into the nucleus, it will bind to key proteins of the cell cycle. However, if p21 remains in the cytoplasm, it will bind with apoptotic proteins which inhibit cell apoptosis and stabilize p21 [17]. A series of researches have revealed that the phosphorylation of p21 stabilizes p21 in the cytoplasm [25]. Cytoplasmic expression of p21, common in human tumors, is related to tumor aggressiveness and prognosis [14, 33]. In our findings, the phosphorylation of p21 and AKT were decreased. P21 localized to the nucleus inhibited the cell cycle. A previous study has reported that cytoplasmic p21 exerts a positive role in promoting tumorigenesis and metastasis by downregulating E-cadherin in vivo [34]. Based on the existing result, it is worth further exploring whether the decreased phosphorylation level of p21 affects PCa cell migration ability.

An important feature of cancer metabolism is the ability to obtain and utilize energy to meet the need for rapid proliferation [35]. Glutamine is an essential nutrient for cancer metabolism. Increased glutamine metabolism drives the ability of biosynthesis in cancers. GLUD1, a key enzyme in mitochondria, has been reported to produce *α*-KG in the liver by catalyzing the deamination of glutamate [36]. However, in cancers, the *α*-KG formation has received increased attention, emphasizing the role of GLUD1 in *α*-KG synthesis [37]. Studies have reported that GLUD1 is overexpressed in lung and colorectal cancers [38, 39]. In breast cancer, GLUD1 expression is significantly higher in ER^+^/HER2^−^ tumors than in other subtypes [40]. In addition, a recent study reported that GLUD1 could regulate redox homeostasis and tumor growth [38]. Moreover, the conversion of glutamate to *α*-KG is accompanied by NADPH production, which is necessary for cell proliferation [41]. In PCa, the expression of GLUD1 was positively related to Gleason scores. A previous study suggested that methylcrotonoyl-CoA carboxylase 2 promotes PCa cell progression and inhibits apoptosis by modulating the GLUD1-P38 MAPK signaling pathway [42]. In the current study, we demonstrated a direct interaction between SIRT4 and GLUD1. However, western blotting results showed that SIRT4 did not affect the expression of GLUD1. This suggests that SIRT4 participates in regulating the posttranslational modifications (PTMS) of GLUD1.

Protein PTMs play a crucial role in tumor progression. A previous study has reported that IDH1 deacetylation that depends on SIRT2 could inhibit CRC and liver metastases [43]. A recent study indicated that SIRT4 promoted the ubiquitination degradation of ANT2, depending on the deacetylation activity in PCa [22]. A study also reported that SIRT3-induced deacetylation of serine hydroxymethyl-transferase2 promotes colorectal carcinogenesis [44]. Another study reported that ADP-ribosylation levels and patterns are related to gene expression and clinical outcomes in ovarian cancers [45]. In the current study, the increased ADP-ribosylation levels affected GLUD1 function in glutamine metabolism in PCa. However, SIRT4 has both deacetylation and ADP-ribosylation activities. Whether deacetylation affects the function of GLUD1 in PCa requires further study.

In conclusion, our results showed that SIRT4 is downregulated in PCa tissues. Low SIRT4 expression was related to PCa progression. Additionally, SIRT4 inhibited PCa cell growth and metastasis by modulating the glutamine metabolism and phosphorylation of p21. In conclusion, these data indicated that SIRT4 may be a novel therapeutic target of PCa.

## Figures and Tables

**Table 1:** The clinical and pathological features of 24 pairs of PCa patients.

		All cases
No. of patients (%)	%	24 (100%)
Age	Years (median, IQR)	70 (67.25–71.25)
PSA	ng/ml (median, IQR)	11.795 (8.005–15.96)

Gleason score		
6	No. of patients (% of total)	5 (20.8%)
7	No. of patients (% of total)	15 (62.5%)
8	No. of patients (% of total)	1 (4.2%)
9	No. of patients (% of total)	3 (12.5%)

## Data Availability

The datasets used and/or analyzed during the current study are available from the corresponding author on reasonable request.

## Supplementary Materials

Supplementary Materials Additional file 1: Table S1: the basic clinical information of PCa patients. Table S2: the list of primers. Table S3: shRNA sequence. Table S4: information of antibodies (DOCX 25 kb). Additional file 2: Supplemental Materials and Methods (DOCX 16 kb).
