# Supplementary material for: Sirtuin 4 Inhibits Prostate Cancer Progression and Metastasis by Modulating p21 Nuclear Translocation and Glutamate Dehydrogenase 1 ADP-Ribosylation
Source: J Oncol. 2022 Jul 7;2022:5498743. doi: 10.1155/2022/5498743 (PMC9283077; doi:10.1155/2022/5498743)
Supplement: Supplementary Materials — Additional file 1: Table S1: the basic clinical information of PCa patients. Table S2: the list of primers. Table S3: shRNA sequence. Table S4: information of antibodies (DOCX 25 kb). Additional file 2: Supplemental Materials and Methods (DOCX 16 kb). [file 5498743.f1.zip › 5498743.f1/Additional files 1.docx]

**Table S1: The list of primers**

| SIRT4 | F: 5’- GGTCAGTGCGGGCATAAA-3’ |
| --- | --- |
|  | R: 5’- TGCTCGAAAGCCTCCATT-3’ |
| p21 | F: 5’-TGTCCGTCAGAACCCATGC-3’ |
|  | R: 5’-AAAGTCGAAGTTCCATCGCTC-3’ |
| GAPDH | F: 5’-GGAGCGAGATCCCTCCAAAAT-3’ |
|  | R: 5’-GGCTGTTGTCATACTTCTCATGG-3’ |

**Table S2: ShRNA sequence**

| SIRT4-RNAi (97759-1) | ccCGATTGCAATACTGAACAT |
| --- | --- |
| SIRT4-RNAi (97760-1) | gaACCCTGACAAGGTTGATTT |
| SIRT4-RNAi (97761-1) | gcGCTTCATCACCCTTTCCAA |

**Table S3: Information of antibodies**

**Primary antibody**

| SIRT4 | proteintech | 66543-1-Ig | 1:10000 |
| --- | --- | --- | --- |
| Actin | proteintech | 66009-1-Ig | 1:5000 |
| CyclinD1 | proteintech | 60186-1-Ig | 1:5000 |
| CDK4 | proteintech | 11026-1-AP | 1:2000 |
| p21 | proteintech | 10225-1-AP | 1:500 |
| p-p21 | Abcam | Ab47300 | 1:1000 |
| AKT | Cell signaling technology | 4691T | 1:1000 |
| p-AKT | Cell signaling technology | 4060T | 1:2000 |
| Flag | Sigma | F3165 | 1:500 |
| GLUD1 | proteintech | 14299-1-AP | 1:2000 |
| GLUD1 | proteintech | 67026-1-Ig | 1:5000 |
| ADP-ribosylation | Cell signaling technology | 83732 | 1:500 |

**Secondary antibody**

| goat anti-mouse IgG | biosharp | BL001A | 1:5000 |
| --- | --- | --- | --- |
| goat anti-rabbit IgG | biosharp | BL003A | 1:5000 |

**Table S4: PCa patients information**

|  | Age (y) | PSA (ng/ml) | Gleason score |
| --- | --- | --- | --- |
| 1 | 61 | 13.10 | 3+3 |
| 2 | 68 | 15.21 | 5+4 |
| 3 | 65 | 6.64 | 3+3 |
| 4 | 75 | 56.45 | 4+4 |
| 5 | 70 | 8.30 | 3+4 |
| 6 | 62 | 29.21 | 3+4 |
| 7 | 63 | 9.84 | 3+3 |
| 8 | 70 | 14.23 | 3+4 |
| 9 | 68 | 5.63 | 3+3 |
| 10 | 70 | 29.04 | 4+3 |
| 11 | 75 | 12.08 | 4+3 |
| 12 | 75 | 9.64 | 3+4 |
| 13 | 71 | 11.51 | 4+5 |
| 14 | 70 | 7.90 | 3+4 |
| 15 | 68 | 9.26 | 4+3 |
| 16 | 62 | 7.64 | 3+3 |
| 17 | 68 | 12.68 | 3+4 |
| 18 | 72 | 81.28 | 4+3 |
| 19 | 75 | 18.21 | 4+5 |
| 20 | 56 | 32.34 | 3+4 |
| 21 | 72 | 6.28 | 4+3 |
| 22 | 71 | 14.07 | 4+3 |
| 23 | 69 | 8.04 | 3+4 |
| 24 | 71 | 4.58 | 3+4 |
