# Supplementary material for: Sirtuin 4 Inhibits Prostate Cancer Progression and Metastasis by Modulating p21 Nuclear Translocation and Glutamate Dehydrogenase 1 ADP-Ribosylation
Source: J Oncol. 2022 Jul 7;2022:5498743. doi: 10.1155/2022/5498743 (PMC9283077; doi:10.1155/2022/5498743)
Supplement: Supplementary Materials — Additional file 1: Table S1: the basic clinical information of PCa patients. Table S2: the list of primers. Table S3: shRNA sequence. Table S4: information of antibodies (DOCX 25 kb). Additional file 2: Supplemental Materials and Methods (DOCX 16 kb). [file 5498743.f1.zip › 5498743.f1/Additional files 2 supplemental Methods.docx]

**Methods**

**5-Ethynyl-2’-deoxyuridine (EdU) assay**

EdU assay was performed using the Cell-Light EdU DNA Cell Proliferation Kit (RiboBio, China). Cells were cultured in 24-well plates and incubated with 50 μM EdU for 2 h incubator . Next, the cells were immobilized in 4% methanol for 30 min and treated with 2 mg/mL glycine for 5 min and 0.5% Triton-100 for 10 min. Each well was washed with PBS three times (10 min each time), and the cells were treated with Apollo reaction reagent for 30 min at room temperature. Finally, the cells were treated with Hoechst-33342 for 30 min and imaged using a fluorescence microscope (Olympus, Japan).

**Immunohistochemistry**

PCa tissue pathological sections and mouse tumor sample sections were incubated with anti-SIRT4 and anti-Ki-67 antibodies overnight at 4 °C. After washing three times, a secondary antibody was added. The cell nuclei were stained with hematoxylin for 10 min, and the expression of SIRT4 and Ki-67 at the protein level was observed using a microscope (Olympus, Japan).

**Flow cytometry assay**

The cell cycle was detected by a Cell Cycle Detection Kit (KeyGEN, China), following the manufacturer’s instructions. Briefly, the cells were washed with PBS and fixed in 70% ice-cold ethanol for 2h at 4 °C and treated with propidium iodide (PI) solution containing 50 μg/ml RNase A. The cell cycle was measured with a FACSCailbur Flow Cytometer (BD Biosciences, USA). Finally, the results were calculated by using Modfit analysis software.
